# Fiscal Impact of Expanded Medicare Coverage for GLP-1 Receptor Agonists to Treat Obesity

**DOI:** 10.1001/jamahealthforum.2025.0905

**Published:** 2025-04-25

**Authors:** Jennifer H. Hwang, Neda Laiteerapong, Elbert S. Huang, Dariush Mozaffarian, A. Mark Fendrick, David D. Kim

**Affiliations:** 1 Department of Medicine, University of Chicago, Chicago, Illinois; 2 Department of Psychiatry and Behavioral Neuroscience, University of Chicago, Chicago, Illinois; 3 Department of Public Health Sciences, University of Chicago, Chicago, Illinois; 4 Food Is Medicine Institute, Friedman School of Nutrition Science and Policy, Tufts University, Boston, Massachusetts; 5 Tufts University School of Medicine, Boston, Massachusetts; 6 Department of Internal Medicine, University of Michigan Medical School, Ann Arbor; 7 Department of Health Management and Policy, School of Public Health, University of Michigan, Ann Arbor

## Abstract

**Importance:**

Despite the clinical benefits of treating obesity and related complications, glucagon-like peptide-1 receptor agonists (GLP-1RAs) are not yet covered by Medicare Part D, partly due to high drug costs. The Biden administration’s proposal to expand Part D coverage underscores the need to assess the balance between fiscal costs and benefits to guide policy decisions.

**Objective:**

To estimate the 10-year net fiscal impact of expanded Medicare coverage for GLP-1RAs for obesity treatment.

**Design, Setting, and Participants:**

In this economic evaluation, a 10-year fiscal impact analysis was conducted between 2026 and 2035 using the validated Diabetes, Obesity, Cardiovascular Disease Microsimulation model. The base-case analysis incorporated a 10% 1-time uptake rate for eligible adults in each new cohort, 40% adherence beyond the first year, and a 10% additional price discount beyond current net prices. A 3-way sensitivity analysis was conducted with varying uptake, adherence, and additional price discounts. The model included current and future Medicare beneficiaries with body mass index (calculated as weight in kilograms divided by height in meters squared) of 30 and higher or 27 and higher with at least 1 obesity-related comorbidity. Data were analyzed from March to December 2024.

**Exposure:**

GLP-1RA therapy.

**Main Outcomes and Measures:**

Total Medicare drug costs for GLP-1RAs for obesity indications not covered by Medicare, long-term health care cost offsets from reduced obesity-related comorbidities, and net fiscal impact.

**Results:**

Among 30 million cumulative Medicare beneficiaries (survey-weighted mean [SE] age, 64.5 [0.4] years; 54.1% female) identified as eligible for new GLP-1RAs for obesity treatment over the next 10 years, the base-case analysis estimated that 3 million would receive treatment. Medicare’s total drug costs were projected at $65.9 billion and health care cost offsets from clinical benefits at $18.2 billion, resulting in net increased spending of $47.7 billion. Three-way sensitivity analysis estimated that higher uptake and adherence would lead to increased health care savings from clinical benefits. However, these savings were estimated to remain less than additional spending on GLP-1RAs and to extensively increase net spending.

**Conclusions and Relevance:**

This economic evaluation estimates that expanded Medicare coverage for GLP-1RAs would increase access and reduce obesity-related comorbidities but impose substantial costs over 10 years. Even with a moderate scenario (5% uptake, 20% adherence, and 30% additional price discount), net spending was still projected to reach $8 billion over a decade, underscoring the need for further price reductions, lower-cost strategies to prevent weight regain, and reductions in spending on low-value care.

## Key Points

**Question:**

What is the projected 10-year fiscal impact on the Medicare program if Part D plans were to cover glucagon-like peptide-1 receptor agonists (GLP-1RAs) for obesity treatment?

**Findings:**

In this economic evaluation of 30 million cumulative Medicare beneficiaries identified as eligible for new GLP-1RAs for obesity treatment over the next 10 years, Medicare’s total projected costs for drug coverage of the GLP-1RAs were $65.9 billion. Health care savings of $18.2 billion were estimated to result in $47.7 billion in net increased spending.

**Meaning:**

Medicare Part D coverage of GLP-1RAs to treat obesity would lead to a substantial net increase in future Medicare spending, and policymakers should leverage a comprehensive range of strategies, including further price reductions resulting from Medicare’s drug price negotiation under the Inflation Reduction Act, lower cost strategies to prevent weight regain, and reductions in spending on unnecessary care.

## Teaser

This economic evaluation estimates the 10-year net fiscal impact of expanded Medicare coverage for glucagon-like peptide-1 (GLP-1) receptor agonists for obesity treatment.

## Introduction

The understanding of obesity has changed since 2003, and in 2013, the American Medical Association officially recognized it as a disease.^1^ The treatment landscape for obesity has also undergone a considerable transformation with the development of highly effective glucagon-like peptide-1 receptor agonists (GLP-1RAs). This new class of antiobesity medications (AOMs) offers substantial clinical benefits, such as sizable weight loss and reductions in obesity-related comorbidities. Other benefits include better glycemic control, reduced recurrent cardiovascular events, improved heart failure symptoms, and alleviated obstructive sleep apnea (OSA).^2,3,4,5,6,7^

The AOM landscape continues to grow, with up to 16 new drugs projected to become available in the next 5 years.^8^ Despite their promise, GLP-1RAs are currently covered by Medicare only for specific indications, such as diabetes (Ozempic, approved in 2017) or cardiovascular disease (CVD; Wegovy, approved in 2024).^9^ Tirzepatide (Zepbound) was recently approved for sleep apnea by the US Food and Drug Administration (FDA) and as of January 2025 is covered by Medicare for this indication.^10,11^ This limitation stems from a historic Medicare Part D provision established in 2003, which excludes drugs labeled for weight loss.^12^ To address this gap, recent legislative and policy initiatives from US Congress and the Centers for Medicare & Medicaid Services (CMS) aim to include AOM coverage under Medicare Part D and Medicaid, redefining these medications as treatments for excess body weight and weight-reduction maintenance.^13,14^

While AOMs provide substantial clinical benefits, they are also costly. For example, GLP-1RAs have an average net cost of $700 to $800 per month after accounting for rebates and discounts.^15^ Long-term compliance also remains low, and weight regain is common following discontinuation.^16^

Prior studies have estimated the resulting budgetary impact, highlighting substantial financial implications.^17,18,19,20,21^ For example, the Congressional Budget Office (CBO) estimated that a bill to allow AOM coverage in Medicare would increase federal spending by $35 billion over 9 years. In comparison, the CMS proposal would increase federal spending by $25 billion due to expanded Part D coverage and $15 billion due to expanded Medicaid coverage over 10 years.^20^

However, estimating the fiscal impacts of the AOM coverage is challenging due to substantial uncertainties in determining the size of the eligible population, long-term treatment uptake and adherence rates, and potential health care cost offsets. To address these gaps, an advanced microsimulation model incorporating nationally representative data was used to provide more accurate clinical and economic estimates. Extensive sensitivity analyses addressed the uncertainty regarding treatment uptake, costs, adherence, and impact on obesity and related comorbidities.

## Methods

### Overview

We projected the 10-year fiscal impact of Medicare Part D coverage of GLP-1RAs (ie, semaglutide and tirzepatide) for obesity treatment using a microsimulation model. Key model parameters include the eligible population, drug uptake, treatment adherence, clinical effectiveness (ie, changes in obesity-related health care spending), and GLP-1RA costs, including potential changes in future prices (eMethods and eTables 1 and 2 in Supplement 1). Key outcomes included direct medication costs, obesity-related health savings, and net spending. All costs were inflated to 2024 US dollars using the health care component of the Personal Consumption Expenditures Price Index.^22^ We did not apply discounting for future costs to be consistent with the standard practice in budget impact analyses.

This study was deemed exempt from review by the University of Chicago institutional review board due to it involving nonhuman participant research. The study adhered to the Consolidated Health Economic Evaluation Reporting Standards (CHEERS) reporting guidelines.^23^

### Diabetes, Obesity, Cardiovascular Disease Microsimulation Model

The Diabetes, Obesity, Cardiovascular Disease Microsimulation model is a validated population disease simulation model that projects individual health trajectories, including the development of obesity (defined as a body mass index [BMI; calculated as weight in kilograms divided by height in meters squared] of ≥30), obesity-related comorbidities, long-term health events (eg, diabetes, myocardial infarction, stroke), and death (eFigure 1 in Supplement 1).^24^ The model incorporates individual-level disease risk based on baseline demographics (eg, age, sex, race and ethnicity), dynamic changes in metabolic risk factors (eg, BMI, blood pressure, total cholesterol, smoking), and an open cohort to estimate long-term health and economic outcomes.

### Eligible Population

**Figure 1.**
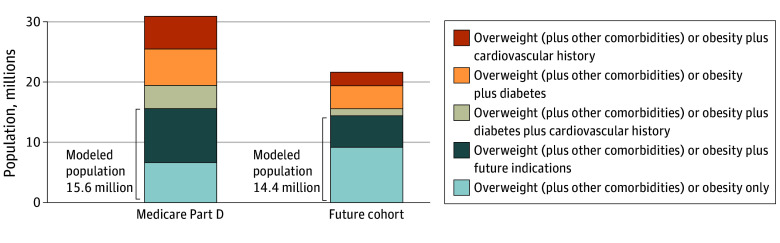
Prevalence of Conditions Indicated for Glucagon-Like Peptide-1 Receptor Agonists Among Medicare Part D Beneficiaries and the Future Cohort Data are from the 2017 to 2020 National Health and Nutrition Examination Survey, including 30 million representative individuals who were current Medicare Part D beneficiaries or who aged into eligibility annually (ie, ≥65 years old) for the future cohort. Overweight was defined as a body mass index (calculated as weight in kilograms divided by height in meters squared) between 27 and 29.9, with at least 1 weight-related comorbidity (eg, prediabetes, hypertension, dyslipidemia, obstructive sleep apnea, or chronic kidney disease). Obesity was defined as a body mass index of 30 or greater. Future indications include chronic kidney disease, obstructive sleep apnea, or osteoarthritis.

Using data from the 2017 to 2020 National Health and Nutrition Examination Survey, the model included 30 million representative individuals who were current Medicare Part D beneficiaries or aged into eligibility annually (ie, ≥65 years old) for future cohorts and met clinical criteria for obesity treatment (ie, BMI ≥30 or BMI ≥27 with ≥1 obesity-related comorbidity, including hypertension, dyslipidemia, chronic kidney disease, OSA, or arthritis) (Figure 1 and eTable 3 in Supplement 1).^25,26^ Individuals with prevalent diabetes or CVD were excluded, as Medicare already provides coverage for these conditions. As dually eligible individuals comprise 17% of Medicare beneficiaries and account for 33% of Medicare spending, we included them to ensure a comprehensive estimate of the fiscal impact.^27,28^

### Uptake and Adherence

Observed GLP-1RA uptake among eligible individuals varies from 2% to 10% annually across reports.^18,20,21,29,30,31^ For this base-case analysis, we applied a 10% 1-time uptake rate for current and future eligible Medicare Part D beneficiaries over the next 10 years, reflecting the upward trend in GLP-1RA use. Empirical evidence suggests that adherence to GLP-1RA therapy in obesity treatment ranges from 27.2% to 73.8% after the first year.^16,32,33,34,35^ Among those who initiated a GLP-1RA therapy, we assumed 100% adherence in the first year, followed by a 40% adherence rate in subsequent years, based on clinical evidence suggesting that individuals adherent at 1 year were more likely to continue treatment.^3^ We assumed that individuals in each cohort who did not initiate GLP-1RA treatment initially, as well as those who discontinued treatment, would not receive GLP-1RAs in the future.

### Clinical Efficacy

Patients receiving AOMs were considered to achieve the mean weight loss and cardiometabolic benefits observed during the first 2 years of clinical trials, including improvements in body weight, systolic blood pressure, diastolic blood pressure, fasting glucose, total cholesterol, high-density lipoprotein cholesterol, and triglycerides (eTable 1 in Supplement 1).^3,36^ No additional weight loss or cardiometabolic risk factor improvements were assumed beyond the first year. The model propagated these improvements into reductions in long-term risks of diabetes and CVD events.

For individuals who discontinued treatment, weight and cardiometabolic risk factors were assumed to revert to baseline levels within a year, followed by risk factor–specific temporal trends in subsequent years.^37,38^ We assumed that benefits diminish with weight regain, reflecting the importance of sustained weight loss.

### Treatment Costs

We estimated the net annual costs of semaglutide and tirzepatide at $8412 and $6236, respectively, reflecting a current 41% and 79% discount from list prices, respectively.^15^ These estimates are derived from commercial net prices reflecting manufacturer discounts and rebates to align with the broader payer landscape. To account for additional drug price discounts in the future from the Inflation Reduction Act (IRA) drug price negotiations and increased competition, we included an additional 10% discount in the base-case analysis, while evaluating additional 20% and 30% discounts in sensitivity analyses.^39^ For example, semaglutide may be selected for Medicare price negotiation under the IRA as early as 2025, with negotiated prices potentially taking effect as early as 2027.^40,41,42^ This year was selected because the IRA negotiation applies to active ingredients, which for semaglutide was approved for diabetes in 2017 (Ozempic), permitting potential cross-indication pricing.^43,44,45^ This approach reflects the earliest eligibility dates for negotiation and the potential alignment of pricing across indications. While the exact discount is uncertain, the first 10 drugs in the IRA program showed a mean 22% reduction in net spending.^45^ Thus, we modeled scenarios with up to an additional 30% discount effective in 2027, based on Medicare guidance and regulatory expectations under the IRA.^39^ For tirzepatide, we applied the same pricing discount, as IRA negotiations are expected to drive down costs and maintain its competitiveness relative to semaglutide.

### Health Care Cost Offsets

We estimated health care cost offsets resulting from improved body weight and cardiometabolic risk factors, which led to the prevention of diabetes, CVD, and related complications. In addition, we incorporated BMI-mediated effects (ie, additional effects on health care costs besides other covariates, including age, sex, race and ethnicity, hypertension, diabetes, and CVD) (eTable 2 in Supplement 1).

### Sensitivity Analysis

To provide a comprehensive assessment of how key factors influence the results, we performed 3-way sensitivity analyses on the impact of varying treatment uptake rates (5%-20%), additional price discounts (10%-30%), and adherence levels (20%-60%) simultaneously. We also evaluated longer-term budget impact over 20- and 30-year horizons. Data were analyzed from March to December 2024.

## Results

### Baseline Characteristics

The analytic sample included 15.6 million current Medicare beneficiaries (current cohort) and an additional 14.4 million individuals in the US who became eligible due to turning 65 years old and meeting obesity-treatment criteria over the next 10 years (future cohort). The survey-weighted mean (SE) age of the analytic sample was 64.5 (0.4) years, 54.1% were female, and the initial mean (SE) BMI was 33.1 (0.3) (Table 1^46^). Among the beneficiaries, prediabetes was the most common comorbidity (81.7%), followed by hypertension (75.5%) and hyperlipidemia (67.5%).

**Table 1.** Baseline Characteristics of Current Medicare Part D Beneficiaries and the Future Cohort With Overweight or Obesity^a^

Characteristic	Weighted mean (SE)		
	Total (N = 1204, representing 30 million)	Current Medicare Part D (n = 675, representing 15.6 million)	Future cohort (n = 529, representing 14.4 million)
Age, y	64.5 (0.4)	69.0 (0.6)	59.6 (0.1)
Sex, %			
Female	54.1 (1.6)	60.1 (1.6)	47.6 (2.9)
Male	45.9 (1.6)	39.9 (1.6)	52.4 (2.9)
Race and ethnicity, %^b^			
Asian and multiracial groups, non-Hispanic	5.7 (0.7)	5.5 (0.9)	6.0 (1.2)
Black, non-Hispanic	10.0 (1.6)	9.7 (1.9)	10.4 (2.1)
Hispanic	11.0 (1.3)	8.8 (1.2)	13.4 (2.0)
White, non-Hispanic	73.2 (2.5)	76.0 (3.0)	70.1 (3.6)
Weight, kg	92.3 (0.8)	89.3 (1.0)	95.5 (1.2)
BMI	33.1 (0.3)	32.8 (0.3)	33.5 (0.4)
Obesity class, %			
Overweight (BMI 27-29.9) with ≥1 comorbidity	33.5 (1.9)	33.6 (2.8)	33.3 (4.2)
Obesity I (BMI 30-34.9)	39.2 (2.6)	40.9 (2.5)	37.3 (5.3)
Obesity II (BMI 35-39.9)	16.8 (1.6)	18.0 (2.2)	15.5 (1.9)
Obesity III (BMI ≥40)	10.6 (1.5)	7.6 (1.4)	13.8 (2.5)
Blood pressure, mm Hg			
Systolic	129.4 (0.7)	130.7 (0.7)	128.0 (1.3)
Diastolic	75.3 (0.3)	73.1 (0.5)	77.7 (0.7)
Fasting blood glucose, mg/dL	105.1 (0.3)	105.8 (0.4)	104.3 (0.5)
Hemoglobin A_1c_, %	5.6 (0.01)	5.7 (0.01)	5.6 (0.01)
Lipid levels, mg/dL			
Total cholesterol	192.7 (2.1)	186.8 (2.6)	199.1 (2.3)
High-density lipoprotein	110.9 (3.1)	54.2 (0.9)	54.2 (1.2)
Low-density lipoprotein	54.2 (0.8)	110.3 (2.3)	123.4 (2.0)
Triglycerides	116.6 (1.6)	113.9 (4.6)	107.7 (4.4)
Mean eGFR, mL/min/1.73 m^2^^c^	82.6 (0.6)	76.5 (1.0)	89.1 (1.0)
Currently smoke, %	10.3 (1.1)	6.6 (1.3)	14.4 (2.6)
Weight-related comorbidity, %^d^			
Chronic kidney disease	9.9 (1)	16.7 (2.0)	2.6 (0.9)
Hyperlipidemia	67.5 (2.2)	73.4 (2.8)	61.0 (4.4)
Hypertension	75.5 (2.2)	80.0 (2.6)	70.8 (3.7)
Metabolic syndrome	25.1 (2.1)	27.3 (2.4)	22.8 (3.4)
Obstructive sleep apnea	22.5 (2)	21.4 (2.8)	23.7 (2.6)
Osteoarthritis	28.2 (2.2)	36.1 (3.3)	19.6 (2.4)
Prediabetes	81.7 (1.7)	83.6 (2.4)	79.6 (3.0)

Abbreviations: BMI, body mass index (calculated as weight in kilograms divided by height in meters squared); eGFR, estimated glomerular filtration rate; NHANES, National Health and Nutrition Examination Survey.

SI conversion factors: To convert glucose to mmol/L, multiply by 0.0555; hemoglobin A_1c_ to proportion of total hemoglobin, multiply by 0.01; cholesterol and lipoproteins to mmol\L, multiply by 0.0259; triglycerides to mmol\L, multiply by 0.0113

^a^ The study cohort was selected from the 2017 to prepandemic 2020 NHANES. The analysis was weighted using NHANES sampling strategies to ensure it accurately reflected the demographic composition of the noninstitutionalized adult population in the US. The initial cohort was identified based on self-reported Medicare status, using specific health insurance variables. To establish the Medicare-eligible cohort for each year, criteria including age, weight, and comorbidities were applied.

^b^ Race and ethnicity data were collected through predefined NHANES categories, with the Asian and multiracial groups, non-Hispanic classification comprising non-Hispanic Asian individuals as well as those from various racial backgrounds, including multiracial participants.

^c^ Calculated using CKD-EPI 2021 equation: eGFR = 142 × min (standardized Scr/K, 1)^α^ × max(standardized Scr/K, 1)^−1.200^ × 0.993^Age^ × 1.012 (if female).

^d^ Chronic kidney disease is defined by an eGFR of 60 mL/min/1.73 m^2^ or lower. Hyperlipidemia is defined as self-reported, receiving treatment, having low-density protein levels more than 160 mg/dL, triglyceride levels more than 150 mg/dL, or high-density lipoprotein levels below 40 mg/dL (<50 mg/dL for female patients). Hypertension is defined by self-report, receiving treatment, or having an average systolic blood pressure of 130 mm Hg or higher or diastolic blood pressure of 80 mm Hg or higher. Metabolic syndrome is defined as meeting at least 3 criteria: elevated waist circumference (≥88 cm for female patients and ≥102 cm for male patients), triglyceride levels of 150 mg/dL or higher, high-density lipoprotein levels lower than 50 mg/dL for female patients or lower than 40 mg/dL for male patients, systolic blood pressure 130 mm Hg or higher or diastolic blood pressure 85 mm Hg or higher, or fasting plasma glucose levels 100 mg/dL or higher. Prediabetes is defined by self-report, fasting plasma glucose levels between 100 and 125 mg/dL, or hemoglobin A_1c_ levels between 5.7% and 6.4%. Obstructive sleep apnea is defined as a STOP-BANG score of 5 or higher.^46^ Osteoarthritis is self-reported.

### Economic Impacts

**Figure 2.**
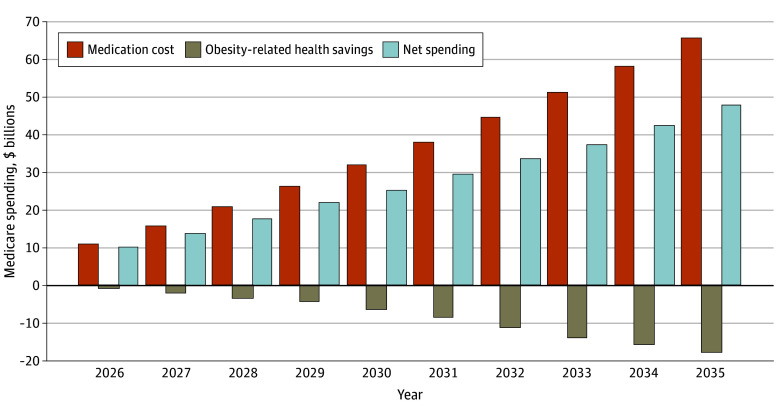
Base-Case Analysis Showing 10-Year Fiscal Impact of Glucagon-Like Peptide-1 Receptor Agonists on Medicare Expenditures for Obesity Treatment This figure presents the projected 10-year fiscal impact of glucagon-like peptide-1 receptor agonists on Medicare expenditures for obesity treatment from 2026 to 2035. The bars represent 3 key components of fiscal impact: medication costs, obesity-related health savings, and net spending.

In the base-case analysis with a 10% 1-time uptake rate of GLP-1RAs (3 million current and future recipients), Medicare’s drug spending on GLP-1RAs was projected to be $65.9 billion over 10 years. Accounting for potential long-term savings due to reducing obesity-related comorbidities ($18.2 billion), net Medicare spending would be $47.7 billion (Table 2, Figure 2, and eFigure 2 in Supplement 1).

**Table 2.** Three-Way Sensitivity Analysis of 10-Year Fiscal Impact^a^

Uptake, %	Price discount, %	$ Billions		
		Adherence 20%	Adherence 40%	Adherence 60%
**Medication cost**				
5	10	21.3	32.9	43.1
	20	19.7	29.9	39.3
	30	17.9	27.6	35.1
10	10	42.6	65.9 (Base case)	88.5
	20	39.5	59.8	79.9
	30	35.8	53.7	71.3
20	10	87.3	131.2	171.1
	20	80.1	119.1	160.1
	30	72.9	107.1	142.9
**Long-term health care cost offsets**				
5	10	−9.9	−11.5	−13.3
	20	−9.9	−11.5	−13.3
	30	−9.9	−11.5	−13.3
10	10	−16.8	−18.2 (Base case)	−18.9
	20	−16.8	−18.2	−18.9
	30	−16.8	−18.2	−18.9
20	10	−37.4	−38.7	−39.5
	20	−37.4	−38.7	−39.5
	30	−37.4	−38.7	−39.5
**Net spending (medication cost + long-term health care cost offsets)**				
5	10	11.4	21.5	29.8
	20	9.8	18.4	26.0
	30	8.0	16.2	21.8
10	10	25.8	47.7 (Base case)	69.5
	20	22.7	41.6	61.0
	30	19.0	35.6	52.4
20	10	49.9	92.6	131.6
	20	42.7	80.5	120.7
	30	35.5	68.4	103.5

^a^ This table presents the 10-year fiscal impact of Medicare Part D coverage of antiobesity medication vs no antiobesity medication coverage for Medicare Part D beneficiaries and the future cohort. This analysis uses a multiway sensitivity framework that considers variations in treatment uptake, price discounts, and adherence levels. Savings (negative values) indicate reductions in budget impact, while costs (positive values) reflect additional expenses. The results underscore the potential economic implications of various treatment scenarios over a 10-year period.

### Three-Way Sensitivity Analysis

Over the 10-year study period, the budget impact of GLP-1RAs on Medicare Part D expenditures was evaluated under varying 1-time uptake rates (5%, 10%, and 20%), additional price discounts (10%, 20%, and 30%), and adherence levels (20%, 40%, and 60%) (Table 2 and eTables 4 and 5 and eFigure 3 in Supplement 1). Medication costs were estimated to increase with higher uptake and adherence levels, but discounts would substantially mitigate the financial impact. For example, at 5% uptake, 10-year medication costs ranged from $17.9 billion (30% additional discount and 20% adherence) to $43.1 billion (10% additional discount and 60% adherence). Similarly, at 20% uptake, medication costs ranged from $72.9 billion (30% additional discount and 20% adherence) to $171.1 billion (10% additional discount and 60% adherence).

Ten-year health care savings were estimated to increase at higher adherence levels. For example, at a 10% uptake, health care savings would increase from −$16.8 billion at 20% adherence to −$18.9 billion at 60% adherence. Higher uptake rates also resulted in more substantial estimated long-term savings, reaching the maximum savings of $39.5 billion at 20% uptake and 60% adherence.

For net spending, at 5% uptake, estimated 10-year net spending ranged from $8.0 billion (30% additional discount and 20% adherence) to $29.8 billion (10% additional discount and 60% adherence). At 20% uptake, estimated net spending ranged from $35.5 billion (30% additional discount and 20% adherence) to $131.6 billion (10% additional discount and 60% adherence). These results highlight that medication costs dominate total expenditures, but greater adherence and uptake are associated with larger long-term health care cost offsets.

### Long-Term Budget Impact Over 20- and 30-Year Horizons

Medication costs are projected to increase steadily from $11.3 billion in 2026 to $65.9 billion by 2035 (undiscounted, year 10), $148.2 billion by 2045 (undiscounted, year 20), and $266.5 billion by 2055 (undiscounted, year 30) (eTable 6 and eFigure 2 in Supplement 1). Obesity-related health care savings are also projected to rise, starting at −$1.0 billion in 2026 and reaching −$18.2 billion, −$44.6 billion, and −$89.3 billion by years 10, 20, and 30, respectively. Net spending is expected to grow from $10.2 billion in 2026 to $47.7 billion in 2035, $103.6 billion in 2045, and $177.3 billion in 2055. Overall, while health care savings are anticipated to grow substantially over time, they are consistently outpaced by the rising costs of GLP-1RAs, increasing net spending across the 30-year horizon.

## Discussion

This analysis of the fiscal impact of expanded Medicare coverage for AOM is distinct because it uses updated net price estimates, considers future price changes, and estimates long-term health care savings from an individual-level health care cost prediction model. These approaches allow us to account for the dynamic, long-term impacts of weight and cardiometabolic risk factor changes on multiple comorbidities and their downstream health care costs. Together, we find that Medicare coverage of GLP-1RAs for obesity will likely result in a substantial fiscal impact, posing considerable affordability challenges to Medicare. For example, total Medicare Part D drug spending on GLP-1RAs could reach $65.9 billion over the next 10 years, assuming a 10% uptake and 40% adherence beyond the first year, and a 10% additional price discount starting in 2027 under the Medicare price negotiation. The costs would be offset by $18.2 billion in obesity-related long-term health care savings, leading to net spending of $47.7 billion over 10 years.

The 3-way sensitivity analysis showed that higher uptakes (at 20%), better adherence (at 60%), and greater additional discounts (at 30%) are estimated increased to health care savings to $39.5 billion. Net spending, however, remains substantial ($103.5 billion) due to the high medication cost ($142.9 billion). Even with conservative estimates (eg, 5% uptake, 30% additional discount, and 20% adherence), net spending is projected to reach $17.9 billion over 10 years, driven by high drug costs. Analyses on longer time horizons (20 and 30 years) consistently suggest net increases in Medicare spending.

Prior studies have provided varying estimates of the fiscal implications of expanding Medicare coverage for GLP-1RAs, reflecting differences in assumptions and methodological approaches (Table 3). Key variations include the size of the eligible population (eg, expanding covered indications and current legislation provisions), assumptions about treatment uptake and adherence, and how medication costs and savings are modeled. For instance, earlier studies, such as those by Baig et al^17^ and Ward et al,^19^ assumed broader eligibility criteria, including all Medicare beneficiaries or those without CVD-specific eligibility. These assumptions led to higher cost projections but limited consideration of current legislative provisions or net medication prices. More recent analyses, such as those by Ippolito et al,^18^ the CBO,^20^ and CMS,^21^ have incorporated narrower eligibility assumptions and updated policy frameworks, resulting in lower cost estimates.^17,18,20,21,47^

**Table 3.** Comparison of Cost Estimation and Budgetary Models for Expanded Medicare Coverage of Obesity Treatments

Variable	Baig et al^17^	Ward et al^19^	Ippolito et al^18^	Congressional Budget Office^20^	CMS^21^	Hwang et al (current study)
Model	Basic cost estimation	The Future Adult Model	Budgetary analysis using Medicare claims data	ICER microsimulation model; the Future Adult Model	Medicare cost estimates	Diabetes, Obesity, Cardiovascular Disease Microsimulation model
Duration	1 y	10-30 y	10 y	2026-2034, with projections to 2044	10 y	10-30 y
Data	2020 Medicare Part D enrollment data	National survey data	Medicare claims data (2016-2019)	Medicare claims data, observational studies, and ICER and the Future Adult modeling	Medicare claims, Medicaid, pricing data	National Health and Nutrition Examination Survey
Eligible population	Adults ≥60 y with obesity	Medicare beneficiaries ≥60 y and adults ≥25 y with obesity or overweight and comorbidities	Medicare beneficiaries with obesity or overweight and comorbidities	Medicare beneficiaries with obesity or overweight and comorbidities	Medicare Part D beneficiaries with obesity only	Medicare Part D beneficiaries with obesity or overweight and comorbidities
Estimated eligible population size	19.5 Million	27.36 Million	10.9 Million	12.5 Million in 2026; 11.9 million in 2034	3.4 Million	15.6 Million in 2026; 30 million in 2035
Intervention	Semaglutide or phentermine/topiramate	Newer weight-loss treatment (not specified), reducing body weight by 20%	GLP-1RA (eg, semaglutide, tirzepatide)	GLP-1RA (eg, semaglutide, tirzepatide)	GLP-1RA (eg, semaglutide, tirzepatide)	GLP-1RA (eg, semaglutide, tirzepatide)
Uptake of treatment	1%-100%, with scenarios at 10% uptake for cost estimation	100%	5% and 10%	2% in the first year (2026), rising to 14% by 2034	10% in the first year, grows by 0.3% annually	10% Annually, with scenarios from 5%-20% annual uptake
Treatment adherence	Not explicitly discussed	100%	40%	35% in year 1; 70% discontinuation for ≥2 y	47.5% Discontinuation within 2 mo	40% Annually, with scenarios from 20%-60% annual adherence
Treatment costs	$670/y (Generic), $13 618/y (brand-name semaglutide)	Not modeled	$8604/y	$5600/y (2026) to $4300/y (2034) after adjustments for beneficiaries’ cost-sharing amounts	Specific drug prices based on pricing comparison	$7324/y (10% additional discount under the Medicare drug price negotiation, effective in 2027)
Health care cost offsets	Not estimated	$176 Billion in 10 y	$970/y per patient; alternative estimates show no offsets	$3.4 Billion (2026-2034); savings grow over time	Not estimated	$18.2 Billion (2026-2035)
Potential fiscal impact	$1.32-$26.8 Billion annually (depending on uptake and drug)	$1-$1.3 Trillion in social benefits over 10 y	$3.1-$6.1 Billion annual Part D increase	$35.5 Billion net federal spending (2026-2034)	$24.8 Billion over 10 y	$47.7 Billion net federal spending (2026-2035)

Abbreviations: CMS, Centers for Medicare & Medicaid Services; GLP-1RA, glucagon-like peptide-1 receptor agonist; ICER, Institute for Clinical and Economic Review.

This study differs in several ways. First, we project a higher number of eligible beneficiaries under the proposed expansion of AOM coverage for obesity treatment by including those with OSA and chronic kidney disease (at the time of the analyses, GLP-1RAs were not approved for treatment for these conditions). For instance, our projected eligible population is larger than the CBO’s and remains higher in future estimates (total of 30 million vs 12.5 million in 2026 and decreased to 11.9 million in 2034). The difference might stem from using National Health and Nutrition Examination Survey data that capture undiagnosed individuals who may not appear in claims records. Second, assumptions about treatment uptake differ substantially. The CBO report assumes gradual uptake, starting at 2% (0.3 million) in 2026 and increasing to 14% by 2034,^20^ while our base case assumes 10% new 1-time uptake. Third, while both analyses consider IRA-related price reductions starting in 2027, we apply a conservative 10% additional discount compared to the CBO’s 32%. Also, while the CBO report accounts for 80% of the net prices after excluding beneficiary out-of-pocket costs, this study does not explicitly model beneficiary cost sharing. Finally, our model provides granular estimates of long-term health care cost offsets by integrating individual-level demographic and health characteristics into the health care cost prediction model. Specifically, we extrapolated trial-based efficacy on weight reduction and associated cardiometabolic improvements to forecast impacts on long-term complications and costs. In contrast to the CBO report that estimated that Medicare coverage for GLP-1RAs would yield $3.4 billion in health care savings and $35.5 billion in net federal spending from 2026 to 2034 (12.5 million Medicare Part D beneficiaries, 2% uptake, and 35% adherence),^20^ the present study projects substantially more considerable health care savings of $18.2 billion and net spending of $47.7 billion from 2026 to 2035 (10% uptake, 10% additional discount, and 40% adherence).

The coverage of GLP-1RAs has important financial implications due to clinical factors: (1) about 40% of Medicare beneficiaries are potentially eligible for treatment and (2) indefinite use of GLP-1RAs may sustain the health benefits over time.^40^ Despite concerns regarding the potential fiscal impact, the Treat and Reduce Obesity Act (TROA), which aims to expand Medicare coverage to include AOMs for obesity, has made legislative progress, passing the US House of Representatives’ Committee on Ways and Means with strong bipartisan support in July 2024.^48^ CMS’s recent reinterpretation of statutory exclusions to allow AOM coverage for obesity treatment projects that 3.3 million Medicare beneficiaries would become eligible, with an estimated 10% initiating treatment, underscoring the potential for considerable access expansion.^49^

The integration of GLP-1RAs into Medicare may lead to several unintended consequences. First, the substantial financial outlay required to cover these medications may necessitate reallocating resources from other high-value treatments, potentially crowding out essential services.^50^ This reallocation could undermine the quality of care provided to Medicare beneficiaries and create disparities in access to effective treatments. Second, while competitive market forces are likely to moderate drug prices, the current regulatory environment may incentivize manufacturers to set higher initial list prices for new drugs.^50^ This practice, driven by the desire to offset anticipated price negotiations under the IRA, could exacerbate Medicare’s financial challenges and increase the overall cost of care.

To help manage the added costs of GLP-1RAs, the Medicare program could prioritize reducing low-value care, such as spending on US Preventive Services Task Force D-recommended services, and explore alternative weight-maintenance strategies like reduced dosing, behavioral therapy, lower-cost medications, or nutrition-focused interventions after achieving maximum weight loss.^51,52,53,54,55^ Since most drug spending in this analysis is driven by weight maintenance, our projections may overestimate costs if these lower-cost strategies are implemented. Empirical data demonstration projects could evaluate clinical and economic feasibility of such approaches.

### Limitations

This study has some limitations. First, while we projected the budget impact of GLP-1RAs under Medicare, there is no certainty regarding critical variables such as cost, uptake, adherence rates, the eligible population, or the clinical efficacy of these drugs in clinical settings. For example, treatment uptake may differ between lower- vs higher-risk individuals, but we do not have enough data to model the differential uptake across population subgroups. Additionally, the potential influence of the IRA, under which Medicare will negotiate drug prices starting in 2027, adds further uncertainty to the future pricing of GLP-1RAs. The negotiated price will be the lesser of the current net price or a percentage of the nonfederal average manufacturer price, but the extent to which this will impact AOM prices remains unclear.

Second, our model likely underestimates health care cost offsets by excluding noncardiometabolic, obesity-related conditions, such as osteoarthritis and certain cancers, which accounted for substantial economic burdens (eg, $403.1 billion for osteoarthritis in 2016).^56^ Although our model indirectly captured these costs using the BMI’s effects on health care costs, modeling these conditions explicitly could provide a more precise estimate of long-term health care savings, possibly further offsetting the fiscal impact of GLP-1RAs under Medicare.

Third, this analysis focuses on the fiscal impact of GLP-1RAs under Medicare Part D and does not account for increased medical costs related to intensive behavioral therapy or older generic AOMs included in TROA. While Medicare currently covers bariatric surgery (Part A) and intensive behavioral therapy (Part B), these options historically have shown low uptake and limited accessibility. Thus, we excluded potential substitution effects from GLP-1RAs to these alternatives due to insufficient data. If more patients opt out of bariatric surgery for GLP-1RAs, the net spending would be smaller than we estimated. However, given the low utilization of existing treatments, its impact would be negligible. Finally, we acknowledge that the modified TROA provision addressing the continuation of AOMs for individuals transitioning to Medicare represents an important area for future exploration. However, uncertainty remains regarding how many enrollees would carry over AOM use from pre-Medicare coverage and maintain therapy after enrollment. Accurate projections would require robust data on uptake and adherence rates in the pre-Medicare population, but these data are currently limited and beyond the scope of this model.

## Conclusions

In this economic evaluation, the potential coverage of GLP-1RAs under Medicare represents a considerable advancement in obesity treatment, but it also poses considerable financial challenges. To ensure that AOMs remain accessible and affordable without compromising patient outcomes, a range of comprehensive policy levers are needed, such as enhancing access to underserved populations, careful assessment of long-term clinical benefits, reducing low-value care, alternative weight maintenance programs, and encouraging further price reductions through increased competition and Medicare’s price negotiation program. Such consorted efforts are crucial to making obesity treatment equitable and sustainable for all Medicare beneficiaries clinically eligible for their use.

## Notes

Supplement 1.
eMethods
**eTable 1.** Key Model Inputs **eTable 2.** Healthcare Spending Prediction Model and Parameters **eTable 3.** Annual and Cumulative Estimates of Medicare Beneficiaries Entering the Cohort, Representing the U.S. Population, 2026-2035 **eTable 4.** Projected 10-Year Cumulative Medication Costs, Obesity-Related Health Savings, and Net Spending for Medicare: Absolute Changes and Relative Ratios Compared to the Base Case **eTable 5.** Three-Way Sensitivity Analysis:10-Year Fiscal Impact Per Medicare Part D Beneficiary **eTable 6.** Base Case Analysis: Projected 30-Year Cumulative Medication Costs, Obesity-Related Health Savings, and Net Spending (in billions) **eFigure 1.** Diabetes, Obesity, Cardiovascular Disease Microsimulation Anti-obesity Medication Model Overview **eFigure 2.** Projected 30-Year Cumulative Medication Costs, Obesity-Related Health Savings, and Net Spending **eFigure 3.** Trends in Absolute Changes Across Uptake, Price Discounts, and Adherence Levels Compared to the Base Case (10% uptake, 10% price discount, 40% adherence)
eReferences

Supplement 2.
Data Sharing Statement

## References

[1] Recognition of obesity as a disease H-440.842. American Medical Association. Accessed August 7, 2024. https://policysearch.ama-assn.org/policyfinder/detail/obesity?uri=%2FAMADoc%2FHOD.xml-0-3858.xml

[2] Davies M, Færch L, Jeppesen OK, ; STEP 2 Study Group. Semaglutide 2·4 mg once a week in adults with overweight or obesity, and type 2 diabetes (STEP 2): a randomised, double-blind, double-dummy, placebo-controlled, phase 3 trial. Lancet. 2021;397(10278):971-984. doi:10.1016/S0140-6736(21)00213-0 33667417

[3] Jastreboff AM, Aronne LJ, Ahmad NN, ; SURMOUNT-1 Investigators. Tirzepatide once weekly for the treatment of obesity. N Engl J Med. 2022;387(3):205-216. doi:10.1056/NEJMoa2206038 35658024

[4] Lincoff AM, Brown-Frandsen K, Colhoun HM, ; SELECT Trial Investigators. Semaglutide and cardiovascular outcomes in obesity without diabetes. N Engl J Med. 2023;389(24):2221-2232. doi:10.1056/NEJMoa2307563 37952131

[5] Kosiborod MN, Abildstrøm SZ, Borlaug BA, ; STEP-HFpEF Trial Committees and Investigators. Semaglutide in patients with heart failure with preserved ejection fraction and obesity. N Engl J Med. 2023;389(12):1069-1084. doi:10.1056/NEJMoa2306963 37622681

[6] Tirzepatide reduced sleep apnea severity by up to nearly two-thirds in adults with obstructive sleep apnea (OSA) and obesity. News release. Lilly. April 17, 2024. Accessed June 20, 2024. https://investor.lilly.com/news-releases/news-release-details/tirzepatide-reduced-sleep-apnea-severity-nearly-two-thirds

[7] Malhotra A, Grunstein RR, Fietze I, ; SURMOUNT-OSA Investigators. Tirzepatide for the treatment of obstructive sleep apnea and obesity. N Engl J Med. 2024;391(13):1193-1205. doi:10.1056/NEJMoa2404881 38912654 PMC11598664

[8] McGrath C. Study: 16 new obesity drugs could come to market in the next 5 years. Healthcare Brew. September 21, 2024. Accessed September 21, 2024. https://www.healthcare-brew.com/stories/2024/09/12/study-new-obesity-drugs

[9] Cubanski J, Neuman T, Sroczynski N, Damico A. A new use for Wegovy opens the door to Medicare coverage for millions of people with obesity. KFF. April 24, 2024. Accessed December 8, 2024. https://www.kff.org/medicare/issue-brief/a-new-use-for-wegovy-opens-the-door-to-medicare-coverage-for-millions-of-people-with-obesity/

[10] FDA approves first medication for obstructive sleep apnea. News release. US Food and Drug Administration. December 20, 2024. Accessed January 2, 2025. https://www.fda.gov/news-events/press-announcements/fda-approves-first-medication-obstructive-sleep-apnea

[11] Eli Lilly's obesity drug gets Medicare coverage for sleep apnea. Reuters. January 9, 2025. Accessed April 3, 2025. https://www.reuters.com/business/healthcare-pharmaceuticals/eli-lillys-obesity-drug-gets-medicare-coverage-sleep-apnea-cnbc-reports-2025-01-08/

[12] Medicare Prescription Drug, Improvement, and Modernization Act of 2003, HR 1, 108th Cong (2003-2004). Pub L No. 108-173. Accessed August 7, 2024. https://www.congress.gov/bill/108th-congress/house-bill/1

[13] Contract Year 2026 policy and technical changes to the Medicare Advantage program, Medicare prescription drug benefit program, Medicare Cost Plan program, and programs of all-inclusive care for the elderly (CMS-4208-P). Centers for Medicare & Medicaid Services. November 26, 2024. Accessed December 26, 2024. https://www.cms.gov/newsroom/fact-sheets/contract-year-2026-policy-and-technical-changes-medicare-advantage-program-medicare-prescription

[14] *Federal Register* documents currently on public inspection. Federal Register. Accessed December 15, 2024. https://www.federalregister.gov/public-inspection/current

[15] Ippolito BN, Levy JF. Estimating the cost of new treatments for diabetes and obesity. AEI Economic Perspectives. September 2023. Accessed March 25, 2025. https://www.aei.org/wp-content/uploads/2023/09/Estimating-the-Cost-of-New-Treatments-for-Diabetes-and-Obesity.pdf

[16] Gleason PP, Urick BY, Marshall LZ, Friedlander N, Qiu Y, Leslie RS. Real-world persistence and adherence to glucagon-like peptide-1 receptor agonists among obese commercially insured adults without diabetes. J Manag Care Spec Pharm. 2024;30(8):860-867. doi:10.18553/jmcp.2024.23332 38717042 PMC11293763

[17] Baig K, Dusetzina SB, Kim DD, Leech AA. Medicare Part D coverage of antiobesity medications—challenges and uncertainty ahead. N Engl J Med. 2023;388(11):961-963. doi:10.1056/NEJMp2300516 36912541

[18] Ippolito B, Levy JF. Expanding Medicare coverage of anti-obesity medicines could increase annual spending by $3.1 billion to $6.1 billion. Health Aff (Millwood). 2024;43(9):1254-1262. doi:10.1377/hlthaff.2024.00356 39146500

[19] Ward AS, Tysinger B, Nguyen PG, Goldman D, Lakdawalla D. Benefits of Medicare coverage for weight loss drugs. University of Southern California Schaeffer Institute. April 18, 2023. Accessed June 21, 2024. https://healthpolicy.usc.edu/research/benefits-of-medicare-coverage-for-weight-loss-drugs/

[20] How would authorizing Medicare to cover anti-obesity medications affect the federal budget? Congressional Budget Office. October 2024. Accessed December 9, 2024. https://www.cbo.gov/publication/60816

[21] Medicare coverage of anti-obesity medications. Assistant Secretary for Planning and Evaluation. November 26.2024. Accessed December 9, 2024. https://aspe.hhs.gov/sites/default/files/documents/127bd5b3347b34be31ac5c6b5ed30e6a/medicare-coverage-anti-obesity-meds.pdf

[22] Using appropriate price indices for analyses of health care expenditures or income across multiple years. Agency for Healthcare Research and Quality. Accessed February 15, 2024. https://meps.ahrq.gov/about_meps/Price_Index.shtml

[23] Husereau D, Drummond M, Augustovski F, ; CHEERS 2022 ISPOR Good Research Practices Task Force. Consolidated Health Economic Evaluation Reporting Standards 2022 (CHEERS 2022) statement: updated reporting guidance for health economic evaluations. BMC Med. 2022;20(1):23. doi:10.1186/s12916-021-02204-0 35022047 PMC8753858

[24] Kim DD, Wang L, Lauren BN, . Development and validation of the US Diabetes, Obesity, Cardiovascular Disease Microsimulation (DOC-M) model: health disparity and economic impact model. Med Decis Making. 2023;43(7-8):930-948. doi:10.1177/0272989X231196916 37842820 PMC10625721

[25] Garvey WT, Mechanick JI, Brett EM, ; Reviewers of the AACE/ACE Obesity Clinical Practice Guidelines. American Association of Clinical Endocrinologists and American College of Endocrinology comprehensive clinical practice guidelines for medical care of patients with obesity. Endocr Pract. 2016;22(suppl 3):1-203. doi:10.4158/EP161365.GL 27219496

[26] Apovian CM, Aronne LJ, Bessesen DH, ; Endocrine Society. Pharmacological management of obesity: an Endocrine Society clinical practice guideline. J Clin Endocrinol Metab. 2015;100(2):342-362. doi:10.1210/jc.2014-3415 25590212

[27] Peña MT, Mohamed M, Burns A, Fuglesten Biniek J, Ochieng N, Chidambaram P. A profile of Medicare-Medicaid enrollees (dual eligibles). KFF. January 31, 2023. Accessed January 1, 2025, 2025. https://www.kff.org/medicare/issue-brief/a-profile-of-medicare-medicaid-enrollees-dual-eligibles/

[28] Seniors & Medicare and Medicaid enrollees. Medicaid.gov. Accessed January 1, 2025. https://www.medicaid.gov/medicaid/eligibility/seniors-medicare-and-medicaid-enrollees.

[29] Kline C, Heinrich A, Holcomb K, Klaisner J, Kuester M. Impact of anti-obesity medication coverage in Medicare Part D. Milliman. February 23, 2024. Accessed June 26, 2024. https://www.milliman.com/-/media/milliman/pdfs/2024-articles/3-6-24_impact-of-covering_anti-obesity-medications-in-medicare-part-d.ashx

[30] KFF Health Tracking Poll May 2024: the public’s use and views of GLP-1 drugs. KFF. May 10, 2024. Accessed June 26, 2024. https://www.kff.org/health-costs/poll-finding/kff-health-tracking-poll-may-2024-the-publics-use-and-views-of-glp-1-drugs/

[31] Weighing the GLP-1 market. Goldman Sachs. April 12, 2024. Accessed June 26, 2024. https://www.goldmansachs.com/pdfs/insights/pages/gs-research/weighing-the-glp1-market/report.pdf

[32] Weiss T, Carr RD, Pal S, . Real-world adherence and discontinuation of glucagon-like peptide-1 receptor agonists therapy in type 2 diabetes mellitus patients in the United States. Patient Prefer Adherence. 2020;14:2337-2345. doi:10.2147/PPA.S277676 33273810 PMC7708309

[33] Palanca A, Ampudia-Blasco FJ, Calderón JM, . Real-world evaluation of GLP-1 receptor agonist therapy persistence, adherence and therapeutic inertia among obese adults with type 2 diabetes. Diabetes Ther. 2023;14(4):723-736. doi:10.1007/s13300-023-01382-9 36847952 PMC10064368

[34] Nguyen H, Dufour R, Caldwell-Tarr A. Glucagon-like peptide-1 receptor agonist (GLP-1RA) therapy adherence for patients with type 2 diabetes in a Medicare population. Adv Ther. 2017;34(3):658-673. doi:10.1007/s12325-016-0470-y 28078541 PMC5350190

[35] Do D, Lee T, Peasah SK, Good CB, Inneh A, Patel U. GLP-1 receptor agonist discontinuation among patients with obesity and/or type 2 diabetes. JAMA Netw Open. 2024;7(5):e2413172. doi:10.1001/jamanetworkopen.2024.13172 38787563 PMC11127113

[36] Wilding JPH, Batterham RL, Calanna S, ; STEP 1 Study Group. Once-weekly semaglutide in adults with overweight or obesity. N Engl J Med. 2021;384(11):989-1002. doi:10.1056/NEJMoa2032183 33567185

[37] Garvey WT, Batterham RL, Bhatta M, ; STEP 5 Study Group. Two-year effects of semaglutide in adults with overweight or obesity: the STEP 5 trial. Nat Med. 2022;28(10):2083-2091. doi:10.1038/s41591-022-02026-4 36216945 PMC9556320

[38] Aronne LJ, Sattar N, Horn DB, ; SURMOUNT-4 Investigators. Continued treatment with tirzepatide for maintenance of weight reduction in adults with obesity: the SURMOUNT-4 randomized clinical trial. JAMA. 2024;331(1):38-48. doi:10.1001/jama.2023.24945 38078870 PMC10714284

[39] Hernandez I, Cousin EM, Wouters OJ, Gabriel N, Cameron T, Sullivan SD. Price benchmarks of drugs selected for Medicare price negotiation and their therapeutic alternatives. J Manag Care Spec Pharm. 2024;30(8):762-772. doi:10.18553/jmcp.2024.24153 38905356 PMC11293770

[40] Hernandez I, Wright DR, Guo J, Shrank WH. Medicare Part D coverage of anti-obesity medications: a call for forward-looking policy reform. J Gen Intern Med. 2024;39(2):306-308. doi:10.1007/s11606-023-08416-9 37715099 PMC10853087

[41] Dickson S, Hernandez I. Drugs likely subject to Medicare negotiation, 2026-2028. J Manag Care Spec Pharm. 2023;29(3):229-235. doi:10.18553/jmcp.2023.29.3.229 36840960 PMC10387900

[42] Johnson M, Kishore S, Nayak RK, Dusetzina SB. The Inflation Reduction Act: how will Medicare negotiating drug prices impact patients with heart disease? Curr Cardiol Rep. 2023;25(6):577-581. doi:10.1007/s11886-023-01878-7 37097432

[43] Wegovy. Package insert. Novo Nordisk; 2022.

[44] Ozempic. Package insert. Novo Nordisk; 2023.

[45] Medicare drug price negotiation program: negotiated prices for initial price applicability year 2026. Centers for Medicare & Medicaid Services. August 15, 2024. Accessed September 12, 2024. https://www.cms.gov/newsroom/fact-sheets/medicare-drug-price-negotiation-program-negotiated-prices-initial-price-applicability-year-2026

[46] STOP-BANG score for obstructive sleep apnea. MDCalc. Accessed March 31, 2025. https://www.mdcalc.com/calc/3992/stop-bang-score-obstructive-sleep-apnea

[47] Ward ZJ, Bleich SN, Cradock AL, . Projected U.S. state-level prevalence of adult obesity and severe obesity. N Engl J Med. 2019;381(25):2440-2450. doi:10.1056/NEJMsa1909301 31851800

[48] Treat and Reduce Obesity Act of 2023, HR 4818, 118th Cong (2023-2024). Accessed June 20, 2024. Accessed March 25, 2025. https://www.congress.gov/bill/118th-congress/house-bill/4818

[49] Sachs R. Proposed rule would expand Medicare and Medicaid coverage for anti-obesity medications. HealthAffairs. December 17, 2024. Accessed December 30, 2024. https://www.healthaffairs.org/content/forefront/proposed-rule-would-expand-medicare-and-medicaid-coverage-anti-obesity-medications

[50] Nagel KE, Ramachandran R, Lipska KJ. Lessons from insulin: policy prescriptions for affordable diabetes and obesity medications. Diabetes Care. 2024;47(8):1246-1256. doi:10.2337/dci23-0042 38536964 PMC11272967

[51] Budros M, Fendrick AM. Levers to reduce use of unnecessary services: creating needed headroom to enhance spending on evidence-based care. Am J Manag Care. 2018;24(8):353-355. 30130031

[52] Kim DD, Daly AT, Koethe BC, . Low-value prostate-specific antigen test for prostate cancer screening and subsequent health care utilization and spending. JAMA Netw Open. 2022;5(11):e2243449. doi:10.1001/jamanetworkopen.2022.43449 36413364 PMC9682424

[53] Schwartz AL, Landon BE, Elshaug AG, Chernew ME, McWilliams JM. Measuring low-value care in Medicare. JAMA Intern Med. 2014;174(7):1067-1076. doi:10.1001/jamainternmed.2014.1541 24819824 PMC4241845

[54] Oronce CIA, Fendrick AM, Ladapo JA, Sarkisian C, Mafi JN. The utilization and costs of Grade D USPSTF services in Medicare, 2007-2016. J Gen Intern Med. 2021;36(12):3711-3718. doi:10.1007/s11606-021-06784-8 33852141 PMC8045442

[55] Kim DD, Hwang JH, Fendrick AM. Balancing innovation and affordability in anti-obesity medications: the role of an alternative weight-maintenance program. Health Aff Sch. 2024;2(6):qxae055. doi:10.1093/haschl/qxae055 38828004 PMC11138958

[56] Waters H, Graf M. The costs of chronic disease in the US. Milken Institute. August 2018. Accessed January 1, 2025. https://milkeninstitute.org/sites/default/files/reports-pdf/ChronicDiseases-HighRes-FINAL_2.pdf

