# Supplementary material for: Fiscal Impact of Expanded Medicare Coverage for GLP-1 Receptor Agonists to Treat Obesity
Source: JAMA Health Forum. 2025 Apr 25;6(4):e250905. doi: 10.1001/jamahealthforum.2025.0905 (PMC12032556; doi:10.1001/jamahealthforum.2025.0905)
Supplement: Supplement 1. — eMethods eTable 1. Key Model Inputs eTable 2. Healthcare Spending Prediction Model and Parameters eTable 3. Annual and Cumulative Estimates of Medicare Beneficiaries Entering the Cohort, Representing the U.S. Population, 2026-2035 eTable 4. Projected 10-Year Cumulative Medication Costs, Obesity-Related Health Savings, and Net Spending for Medicare: Absolute Changes and Relative Ratios Compared to the Base Case eTable 5. Three-Way Sensitivity Analysis:10-Year Fiscal Impact Per Medicare Part D Beneficiary eTable 6. Base Case Analysis: Projected 30-Year Cumulative Medication Costs, Obesity-Related Health Savings, and Net Spending (in billions) eFigure 1. Diabetes, Obesity, Cardiovascular Disease Microsimulation Anti-obesity Medication Model Overview eFigure 2. Projected 30-Year Cumulative Medication Costs, Obesity-Related Health Savings, and Net Spending eFigure 3. Trends in Absolute Changes Across Uptake, Price Discounts, and Adherence Levels Compared to the Base Case (10% uptake, 10% price discount, 40% adherence) eReferences [file jamahealthforum-e250905-s001.pdf]

Supplemental Online Content

Hwang JH, Laiteerapong N, Huang ES, Mozaffarian D, Fendrick AM, Kim DD. Fiscal impact of expanded Medicare coverage for GLP-1 receptor agonists to treat obesity. *JAMA Health Forum*. 2025;6(4):e250905. doi:10.1001/jamahealthforum.2025.0905

eMethods
eTable 1. Key Model Inputs
eTable 2. Healthcare Spending Prediction Model and Parameters
eTable 3. Annual and Cumulative Estimates of Medicare Beneficiaries Entering the Cohort, Representing the U.S. Population, 2026-2035
eTable 4. Projected 10-Year Cumulative Medication Costs, Obesity-Related Health Savings, and Net Spending for Medicare: Absolute Changes and Relative Ratios Compared to the Base Case
eTable 5. Three-Way Sensitivity Analysis:10-Year Fiscal Impact Per Medicare Part D Beneficiary
eTable 6. Base Case Analysis: Projected 30-Year Cumulative Medication Costs, Obesity-Related Health Savings, and Net Spending (in billions)
eFigure 1. Diabetes, Obesity, Cardiovascular Disease Microsimulation Anti-obesity Medication Model Overview
eFigure 2. Projected 30-Year Cumulative Medication Costs, Obesity-Related Health Savings, and Net Spending
eFigure 3. Trends in Absolute Changes Across Uptake, Price Discounts, and Adherence Levels Compared to the Base Case (10% uptake, 10% price discount, 40% adherence)
eReferences

This supplemental material has been provided by the authors to give readers additional information about their work.

© 2025 Hwang JH et al. *JAMA Health Forum*.

eMethods

1.1.The Diabetes, Obesity, Cardiovascular Disease Microsimulation (DOC-M) model

The Diabetes, Obesity, Cardiovascular Disease Microsimulation (DOC-M) model is a validated individual-level, health-state transition model programmed in R Statistical Software (version 4.1.0; R Core Team 2021).[1]

The model utilized data from the National Health and Nutrition Examination Survey (NHANES) from 1999 to 2016. These data included demographic, cardiometabolic risk factors and related health conditions to provide estimates to predict annual risks of obesity, diabetes, first atherosclerotic cardiovascular disease (ASCVD) and second ASCVD. The annual incidence of diabetes was estimated using the Framingham Offspring Study 8-year risk prediction model based on systolic blood pressure (SBP), fasting blood glucose, body mass index (BMI), high-density lipoprotein (HDL-C), triglycerides, history of hypertension treatment, and parental history of diabetes.[2] The first ASCVD event was estimated using the American College of Cardiology/American Heart Association (ACC/AHA) 10-year ASCVD risk equation.[3] For the second ASCVD, the Framingham Heart Study coronary risk model was used to estimate 2-year risk based on age, SBP, total cholesterol, smoking status, and diabetes status.[4]

For mortality estimates, the model extracted cause-specific mortality data from the 2012-2016 CDC Wonder data.[5] These data included information on the underlying cause of death and demographic data, stratified by age groups, sex, and four race and ethnicity groups. The model assumed that cause-specific mortalities (e.g., those from ischemic heart disease, stroke, and diabetes) were only applicable to individuals with the respective conditions (CVD history, first or second CVD, and diabetes) rather than the general population.

The model also effectively tracks rates of obesity (BMI ≥30 kg/m²). It is achieved by dynamically tracking the variations in BMI for each individual, taking into account their age, sex, and race and ethnicity to provide U.S. representative estimates. Based on health outcomes, the model estimates quality-adjusted life years (QALYs) and health care costs, providing a detailed individual-level analysis.

Overall, the model enables to evaluate various health policies and interventions by projecting obesity, diabetes, and CVD-related health outcomes, along with their associated complications, health-related quality of life and health care costs. The source code for the original DOC-M is available at https://github.com/food-price/DOC-M-Model-Development-and-Validation.

1.2 Time Horizon

We assessed the 10-year fiscal impact on Medicare if Part D prescription drug coverage expands to include glucagon-like peptide-1 receptor agonists (GLP-1RAs) for obesity treatment. Scenario analyses also included extended projections over 20- and 30-year periods.

1.3 Projection of Weight and Cardiometabolic Risk Factor Changes

We utilized data from current clinical trials to extrapolate changes in weight and related cardiometabolic risk factors, including SBP and diastolic blood pressure (DBP), fasting blood glucose, total cholesterol, HDL-C, and triglycerides, aiming to understand how changes in these cardiometabolic risk factors due to GLP-1RAs impact long-term health outcomes.[6-9] The treatment-induced weight reduction and changes in cardiometabolic risk factors were calibrated to reflect the intention-to-treat analysis of relative changes from baseline, considering the proportion of individuals who discontinued treatment.

© 2025 Hwang JH et al. *JAMA Health Forum*.

Given the lack of long-term data on weight and cardiometabolic risk factor changes and considering that trials exceeding 52 weeks did not demonstrate substantial changes, we have presumed that weight and cardiometabolic risk factors would not further improve after the first year.[10-12] Therefore, we maintained the initial changes achieved with GLP-1RAs throughout the treatment period in our projections.

1.4 Change in Weight and Cardiometabolic Risk Factors after Treatment Discontinuation
Projection of weight regain after an individual discontinued treatment was modeled based on the clinical trials by Wilding et al. and Aronne et al.[8,10,12] In a STEP-1 extension analysis led by Wilding et al., semaglutide led to a mean weight loss of 17.3% over 68 weeks, which decreased to a net loss of 5.6% at week 120 after treatment cessation, reflecting a significant weight regain and a reversion of cardiometabolic gains.[10] Similarly, an 88-week study by Aronne et al. found that participants who discontinued tirzepatide treatment after an initial 36 weeks experienced 14% weight regain after the treatment discontinuation.[12] Based on these findings, individuals were assumed to revert to their baseline weight and cardiometabolic risk factors by the end of the second year following cessation of treatment. Individuals adhering to lifestyle modifications only followed natural trends in weight and cardiometabolic risk factors after the discontinuation.

1.5 Probability of Treatment Discontinuation
Observational studies report a wide range of adherence to GLP-1RA therapy in obesity treatment, with rates varying from 27.2% to 73.8% after the first year.[13-17] However, uncertainty remains around long-term adherence, particularly among Medicare beneficiaries, due to the limited evidence available for this population. For our base case analysis, we assumed a 40% continuation rate beyond the first year (i.e., a 60% discontinuation rate within the first year, largely due to the titration period). Discontinuers were assumed to contribute costs equivalent to 12 monthly refills in the year they initiated treatment. For those completing the first year of GLP-1RA therapy, we assumed no further discontinuation, continuing therapy over the entire 10-year period.

1.6 Estimated Costs
1) Healthcare Cost Estimation:
We estimated annual healthcare costs for individuals based on age, gender, race and ethnicity, BMI, and health conditions, including diabetes, hypertension, and CVD from the 2016-2020 Medical Expenditure Panel Survey (MEPS). The DOC-M model employed a two-stage estimation process: a logit model to predict the probability of healthcare expenditures, followed by a generalized linear model with a log link and gamma distribution to ensure nationally representative cost estimations by incorporating MEPS design and weights.[18] This approach allowed us to calculate the expected annual healthcare spending per individual, reflecting demographic and health changes, with all costs adjusted to 2024 dollars using the health care component of the Personal Consumption Expenditures price index.[19]

2) Treatment costs
In our analysis, the estimated annual net costs for semaglutide and tirzepatide were $8412 and $6236, respectively, representing discounts of 41% and 79% from their list prices, as per recent studies.[20] With equal usage assumed between the two drugs, the average annual net cost for AOMs was calculated to be $7324. The Inflation Reduction Act (IRA) of 2022 allows CMS to

© 2025 Hwang JH et al. *JAMA Health Forum*.

negotiate drug prices, potentially impacting the cost of AOMs like semaglutide starting in 2027. Although the specific effects of price negotiations are uncertain, our projections incorporate an additional 10% discount on current net prices, starting in 2027, based on recent estimates.[21] While tirzepatide is not expected to enter price negotiations until 2030, competitive market dynamics could lower semaglutide prices to remain competitive.

1.7 Validation of Outcomes

The DOC-M was validated by comparing the model output of relative percent weight change from baseline with reported intention-to-treat values. Additionally, the model was validated for changes in six cardiometabolic risk factors, including SBP and DBP, fasting blood glucose, total cholesterol, HDL-C, and triglycerides. This comparison ensured that the model accurately reflected the expected outcomes based on clinical trial data and real-world evidence. The validation process provided confidence in the model's ability to project long-term health outcomes and economic implications accurately.

1.8 Addressing Uncertainty

The DOC-M model is constructed to address various forms of uncertainty: stochastic, which captures the random variability in individual outcomes; parameter, dealing with the precision of model inputs; sampling, reflecting the representativeness of the cohort; and imputation, compensating for any missing data.[18] To mitigate stochastic uncertainty, the model can simulate an individual many times and take the average of these simulations. It also accounts for parameter uncertainty by integrating a variety of input parameters across numerous simulations. For example, a ten-fold replication of individuals, coupled with a thousand Monte Carlo simulations, leads to a significant number of total runs. The model also adjusts for sampling variation by using weighted averages to better estimate population outcomes. A modified version of Rubin's rule is employed to combine variance within and between simulations, providing a comprehensive measure of uncertainty in the results.[22,23]

© 2025 Hwang JH et al. *JAMA Health Forum*.

eTable 1. Key Model Inputs

**Treatment efficacy**			
**Study Arms**	**Semaglutide 2.4mg**	**Tirzepatide 15mg**	**Average treatment effect**
**Clinical Trial**	**STEP-1**[8]	**SURMOUNT-1**[6]	
	**Mean (SE)**	**Mean (SE)**	**Mean (SE)**
**Mean percent obesity loss, %**	−12.4 (0.5)	−17.8 (0.8)	-15.1 (0.6)
**SBP, mmHg**	–5.1 (0.6)	–6.4 (0.7)	-5.8 (0.6)
**DBP, mmHg**	–2.4 (0.4)	–3.6 (0.5)	-3.01 (0.5)
**Fasting glucose, mg/dL**	–7.9 (0.6)	–11.5 (0.7)	-9.7 (0.7)
**Total cholesterol, % change in mg/dL**	−3.0 (0.8)	–6.3 (0.9)	-4.7 (0.9)
**HDL, % change in mg/dL**	4.0 (0.8)	8.4 (1.1)	6.2 (0.9)
**Triglycerides, % change in mg/dL**	−15 (1.5)	–25.1 (1.9)	-20.05 (1.7)

**Disease-specific incidence and mortality**[18]			
**Parameter**	**Model or Probabilities**	**Distribution**	**Source**
Developing type 2 diabetes	Framingham Offspring Study 8-year diabetes risk model	Deterministic[b]	Wilson et al (2007)[2]
First ASCVD	ACC/AHA 10-year ASCVD risk model	Deterministic[b]	Goff et al (2014)[3]
% CHD vs. Stroke	Sex-race-specific values (47.4-64.0% vs. 36.0-52.6%)]	N/A	Benjamin et al (2018)[24]
Second ASCVD	Framingham Heart Study 2-year risk model for second CHD	Deterministic[b]	D'Agostino et al (2000)[4]
% CHD vs. Stroke	Sex-race-specific values (58.0-73.2% vs. 26.8-42.0%)	N/A	Benjamin et al (2018)[24]
Disease-specific mortality	CDC Wonder cause-specific mortality by age, sex, race/ethnicity groups	Beta	CDC (2018)[25]
**Probabilities among individuals undergoing RVSC**[18]			
% receiving RVSC	67.3%	Normal[b]	Lubetkin et al (2005)[26]
% CABG vs. PCI among RVSC	28.9% vs. 71.1%	Beta	Davies et al (2015)[27]

© 2025 Hwang JH et al. *JAMA Health Forum*.

Death from CABG vs. PCI	1.8% vs. 2.1%		Beta	Davies et al (2015)[27]
**Costs, 2024 US $[c]**				
**Annual treatment costs**				
Semaglutide	8412	NA	NA	Ippolito et al[20]
Tirzepatide	6236	NA	NA	Ippolito et al[20]
Average Cost	7324	NA	NA	NA
**Health Care Costs[18]**				
Annual health care cost	Health care cost prediction model	NA	Normal	Kim et al. (2023)[18]
**Event-specific Costs**				
CHD	11644	NA	Gamma	CMS (2019)[28]
Stroke	18561	NA	Gamma	CMS (2019)[28]
CABG	51686	NA	Gamma	CMS (2019)[28]
PCI	21442	NA	Gamma	CMS (2019)[28]

Data are mean and standard error.

The distribution was based on regression coefficients.

Abbreviations: ASCVD, atherosclerotic cardiovascular disease; CABG, coronary artery bypass surgery; CHD, coronary heart disease; DBP, diastolic blood pressure; HDL, high-density lipoprotein; PCI, percutaneous coronary intervention; RVSC, revascularization; SBP, systolic blood pressure; SE, standard error.

© 2025 Hwang JH et al. *JAMA Health Forum*.

eTable 2. Healthcare Spending Prediction Model and Parameters

Predictor	Mean annual healthcare expenditures, $	Standard Error
**Age after 40, each year [i.e., age 40 = 0]**	135.05	12.31
**Female**	1656.73	264.67
**Changes in BMI from BMI 28, each kg/m2[e.g., BMI 25 = -3; BMI 30=2]**	88.58	23.61
**Male**	0.00	0.00
**Non-Hispanic White**	0.00	0.00
**Non-Hispanic Black**	-1994.99	387.42
**Hispanic**	-2770.30	367.84
**Non-Hispanic Other**	-2719.10	465.31
**Hypertension**	2394.29	288.49
**Diabetes**	5718.88	388.29
**Cardiovascular disease history**	6006.72	341.93

We constructed the two-part model with a logit model in the first stage to estimate the probability of incurring any healthcare expenditures, followed by a generalized linear model (GLM) with a log link and a gamma distribution to model healthcare expenditures among those who have any expenditures.

Baseline healthcare expenditures were calculated for individuals aged 40 years, male, non-Hispanic White, with a BMI of 28 and no clinical conditions (e.g., no hypertension, diabetes, or cardiovascular disease). The marginal effects indicate the average change in healthcare expenditures associated with a one-unit increase in each predictor variable, relative to the baseline. For instance, keeping all other predictors constant (i.e., age 40, male, non-Hispanic White, BMI 28, and no clinical conditions), a one-year increase in age (e.g., from 40 to 41) would result in an estimated annual increase of $135 in healthcare expenditures.

Abbreviations: BMI, body mass index

© 2025 Hwang JH et al. *JAMA Health Forum*.

eTable 3. Annual and Cumulative Estimates of Medicare Beneficiaries Entering the Cohort, Representing the U.S. Population, 2026-2035

Year	Number of U.S. Representative Population (millions)	Number of Cumulative U.S. Representative Population (millions)
2026	15.6	15.6
2027	1.2	16.8
2028	1.5	18.3
2029	1.2	19.6
2030	1.2	20.8
2031	1.8	22.6
2032	2.4	25.0
2033	1.2	26.2
2034	1.5	27.8
2035	2.3	30.0
Total	30.0	30.0

Note: The initial cohort includes individuals aged 65 and older, as well as Medicare Part D beneficiaries entering in the first year. In subsequent years, the entering cohort comprises individuals turning 65, resulting in a smaller cohort size compared to the first year.

© 2025 Hwang JH et al. *JAMA Health Forum*.

eTable 4. Projected 10-Year Cumulative Medication Costs, Obesity-Related Health Savings, and Net Spending for Medicare: Absolute Changes and Relative Ratios Compared to the Base Case

Absolute Changes Compared to the Base Case (in billion dollars)				
**Uptake (%)**	**Price Discount (%)**	**Adherence (%)**		
		**20**	**40**	**60**
**5**	**10**	−36.3	−26.2	−17.9
**5**	**20**	−37.9	−29.3	−21.7
**5**	**30**	−39.7	−31.5	−25.9
**10**	**10**	−21.9	Base Case	21.8
**10**	**20**	−25	−6.1	13.3
**10**	**30**	−28.7	−12.1	4.7
**20**	**10**	2.2	44.9	83.9
**20**	**20**	−5	32.8	73
**20**	**30**	−12.2	20.7	55.8
**Relative Changes Compared to the Base Case (%)**				
**Uptake (%)**	**Price Discount (%)**	**Adherence (%)**		
		**20**	**40**	**60**
**5**	**10**	↓76%	↓55%	↓38%
**5**	**20**	↓79%	↓61%	↓45%
**5**	**30**	↓83%	↓66%	↓54%
**10**	**10**	↓46%	Base Case	↑46%
**10**	**20**	↓52%	↓13%	↑28%
**10**	**30**	↓60%	↓25%	↑10%
**20**	**10**	↑5%	↑94%	↑176%
**20**	**20**	↓10%	↑69%	↑153%
**20**	**30**	↓26%	↑43%	↑117%

The table presents absolute changes and relative ratios compared to the base case scenario, measured over a 10-year cumulative period.

The base case assumes 10% uptake, 10% price discount, and 40% adherence, resulting in a cumulative net spending of 47.7 billion dollars.

The absolute changes (in billions of dollars) represent the difference in net spending compared to the base case. Negative values indicate savings (a decrease in spending), while positive values indicate additional spending. For example, at 5% uptake, 10% price discount, and 20% adherence, the absolute change is -36.3 billion dollars, indicating savings of 36.3 billion compared to the base case. In contrast, at 20% uptake, 10% price discount, and 60% adherence, the absolute change is 83.9 billion dollars, reflecting an increase in spending.

© 2025 Hwang JH et al. *JAMA Health Forum*.

The relative change illustrate how costs change relative to the base case, with directional arrows denoting the direction of change. A down arrow (↓) indicates a saving direction (lower costs compared to the base case), while an up arrow (↑) signifies a spending direction (higher costs compared to the base case). For example, at 10% uptake, 20% price discount, and 60% adherence, the relative change is ↑28%, meaning costs are 28% higher than the base case.

© 2025 Hwang JH et al. *JAMA Health Forum*.

eTable 5. Three-Way Sensitivity Analysis:10-Year Fiscal Impact Per Medicare Part D Beneficiary

A: Medication cost, $				
**Uptake (%)**	**Price Discount (%)**	**Adherence (20%)**	**Adherence (40%)**	**Adherence (60%)**
5	10	708	1094	1432
	20	653	993	1305
	30	594	918	1165
10	10	1415	2188 (Base case)	2939
	20	1313	1986	2654
	30	1190	1785	2369
20	10	2900	4359	5683
	20	2662	3958	5320
	30	2423	3558	4749
**B: Long-term Health Care Cost Offsets, $**				
**Uptake (%)**	**Price Discount (%)**	**Adherence (20%)**	**Adherence (40%)**	**Adherence (60%)**
5	10	−329	−381	−441
	20	−329	−381	−441
	30	−329	−381	−441
10	10	−558	−603 (Base case)	−629
	20	−558	−603	−629
	30	−558	−603	−629
20	10	−1243	−1284	−1311
	20	−1243	−1284	−1311
	30	−1243	−1284	−1311
**C: Net spending (=A + B), $**				
**Uptake (%)**	**Price Discount (%)**	**Adherence (20%)**	**Adherence (40%)**	**Adherence (60%)**
5	10	380	713	991
	20	324	612	865
	30	266	537	725
10	10	857	1585 (Base case)	2309
	20	755	1383	2025
	30	632	1182	1740
20	10	1657	3075	4372
	20	1418	2674	4009
	30	1180	2273	3438

© 2025 Hwang JH et al. *JAMA Health Forum*.

eTable 6. Base Case Analysis: Projected 30-Year Cumulative Medication Costs, Obesity-Related Health Savings, and Net Spending (in billions)

Year	Direct Medication Cost ($)	Obesity-related Health Savings ($)	Net Spending ($)
2026	11.3	−1.0	10.2
2027	16.0	−2.1	13.9
2028	21.2	−3.5	17.7
2029	26.5	−4.6	21.9
2030	32.0	−6.7	25.3
2031	38.1	−8.8	29.3
2032	44.9	−11.3	33.6
2033	51.4	−14.2	37.2
2034	58.3	−16.1	42.2
2035	65.9	−18.2	47.7
2036	72.9	−19.7	53.2
2037	78.8	−21.9	56.9
2038	85.3	−23.7	61.6
2039	97.2	−26.8	70.4
2040	102.8	−28.9	73.8
2041	111.6	−31.6	79.9
2042	119.4	−34.6	84.8
2043	127	−37.3	89.7
2044	139.3	−41.6	97.7
2045	148.2	−44.6	103.6
2046	158.3	−48.4	109.9
2047	169.4	−52.2	117.1
2048	175.8	−54	121.8
2049	187.6	−58.2	129.4
2050	200.2	−62.6	137.6
2051	209.8	−66.5	143.3
2052	222.9	−70.9	152
2053	234.5	−75.5	159
2054	247.6	−81.2	166.4
2055	266.5	−89.3	177.3

Note: This analysis is based on an open-cohort model, which assumes that new Medicare Part D beneficiaries will become eligible for anti-obesity medication (AOM) coverage over the next 30 years.

© 2025 Hwang JH et al. *JAMA Health Forum*.

eFigure 1. Diabetes, Obesity, Cardiovascular Disease Microsimulation Anti-obesity Medication Model Overview

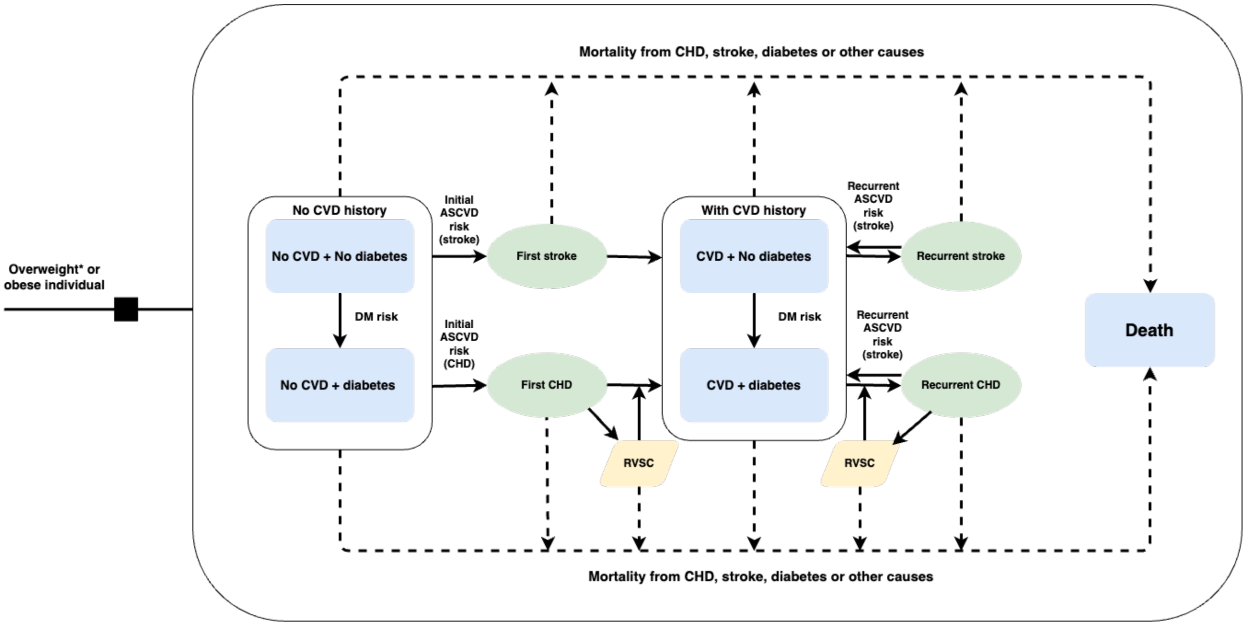

The Diabetes, Obesity, Cardiovascular Disease Microsimulation (DOC-M) model

The figure has been adapted from the original framework developed by Kim et al. to suit the specific context of our study. Individuals either receive lifestyle modifications or anti-obesity medication (AOM) in conjunction with lifestyle modification. Decision nodes (black squares) indicate treatment decisions, and chance nodes (black circles) represent possible outcomes.

The Markov model incorporates five health states—depicted as blue rectangles—where individuals may reside over time, along with potential cardiovascular disease events—represented as green circles—that occur annually. Solid arrows depict transitions between health states and events, while dotted arrows indicate mortality linked to specific causes or events. The simulation continuously updates everyone's health metrics, dictating their journey through various health states and the probability of events.

Overweight is defined as a BMI of 27–29 kg/m² with at least one weight-related comorbidity.

Abbreviations: ASCVD, atherosclerotic cardiovascular disease; CHD, coronary heart disease; CVD, cardiovascular disease; LM, lifestyle modification; RVSC, revascularization (including coronary artery bypass surgery and percutaneous coronary intervention).

© 2025 Hwang JH et al. *JAMA Health Forum*.

eFigure 2. Projected 30-Year Cumulative Medication Costs, Obesity-Related Health Savings, and Net Spending

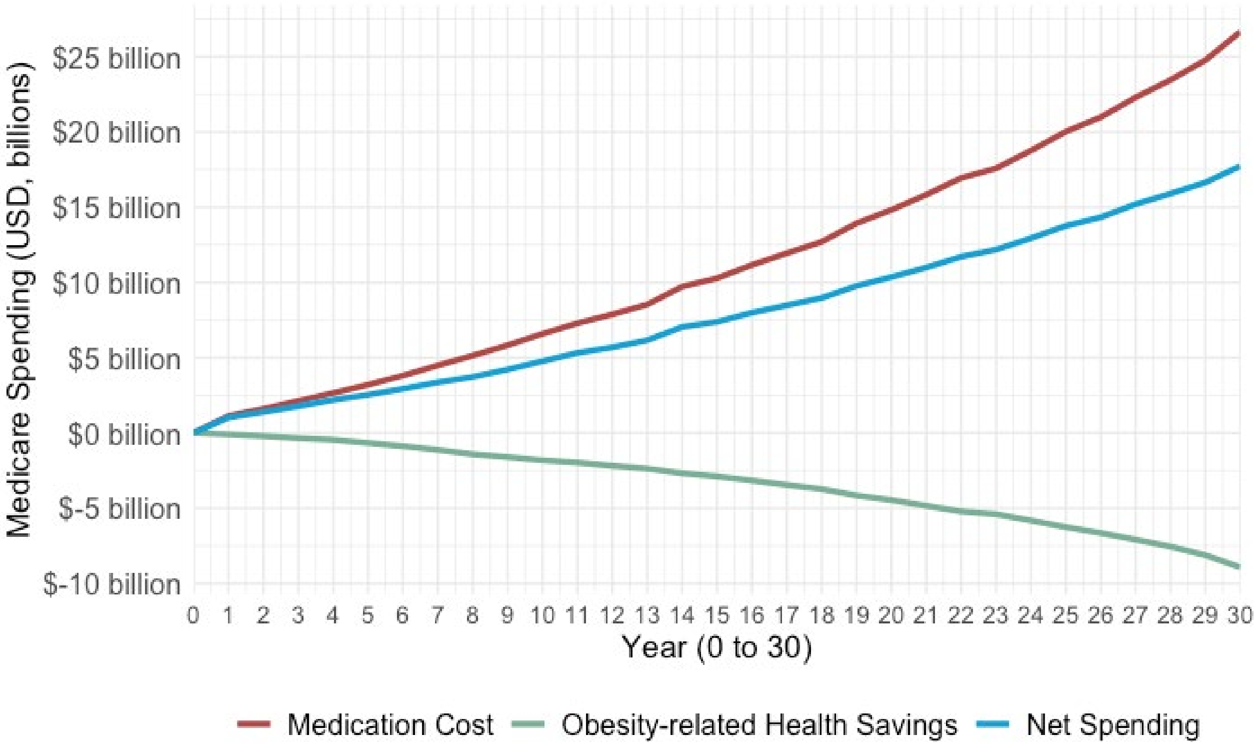

Note: As shown in the figure, the projected costs for anti-obesity medications (AOMs) are expected to increase significantly over time, rising from $65.9 billion in 2035 (Year 10) to $148.2 billion in 2045 (Year 20) and $266.5 billion in 2055 (Year 30).

At the same time, long-term health care cost offsets—driven by reductions in obesity-related complications—are also projected to grow, from -$18.2 billion in 2035 (Year 10) to -$44.6 billion in 2045 (Year 20) and -$89.3 billion in 2055 (Year 30). As a result, the net costs (AOM costs minus health care cost offsets) are expected to decrease over time, from $47.7 billion in 2035 (Year 10) to $103.6 billion in 2045 (Year 20) and $177.3 billion in 2055 (Year 30).

© 2025 Hwang JH et al. *JAMA Health Forum*.

eFigure 3. Trends in Absolute Changes Across Uptake, Price Discounts, and Adherence Levels Compared to the Base Case (10% uptake, 10% price discount, 40% adherence) (Based on eTable 5)

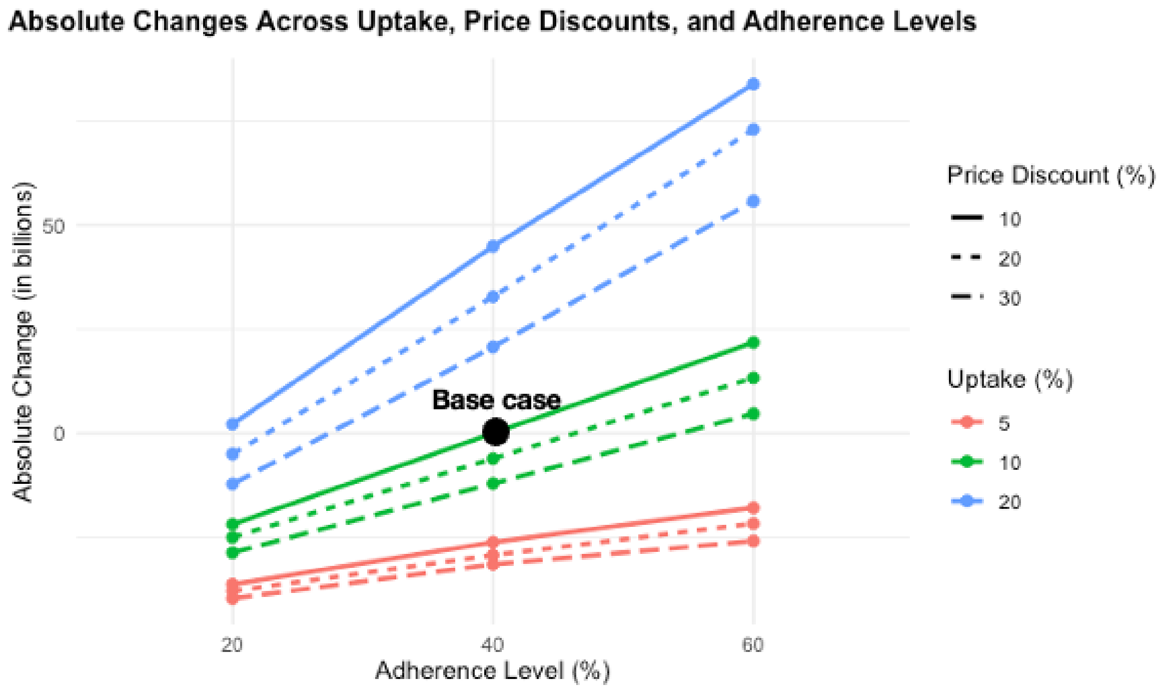

This figure illustrates the absolute changes in projected net spending (in billions of dollars) across different adherence levels (20%, 40%, 60%), uptake rates (5%, 10%, 20%), and price discount levels (10%, 20%, 30%).

© 2025 Hwang JH et al. *JAMA Health Forum*.

eReferences

1. *R: A language and environment for statistical computing*. Version 4.2.0. R Foundation for Statistical Computing; 2021. https://www.R-project.org
2. Wilson PWF, Meigs JB, Sullivan L, Fox CS, Nathan DM, D'Agostino RB, Sr. Prediction of Incident Diabetes Mellitus in Middle-aged Adults: The Framingham Offspring Study. *Archives of Internal Medicine*. 2007;167(10):1068-1074. doi:10.1001/archinte.167.10.1068
3. Goff DC, Lloyd-Jones DM, Bennett G, et al. 2013 ACC/AHA Guideline on the Assessment of Cardiovascular Risk. *Circulation*. 2014;129(25_suppl_2):S49-S73. doi:doi:10.1161/01.cir.0000437741.48606.98
4. D'Agostino RB, Russell MW, Huse DM, et al. Primary and subsequent coronary risk appraisal: New results from the Framingham study. *American Heart Journal*. 2000/02/01/ 2000;139(2, Part 1):272-281. doi:https://doi.org/10.1016/S0002-8703(00)90236-9
5. Centers for Disease Control and Prevention: Underlying Cause of Death. 2018;
6. Jastreboff AM, Aronne LJ, Ahmad NN, et al. Tirzepatide Once Weekly for the Treatment of Obesity. *New England Journal of Medicine*. 2022/07/21 2022;387(3):205-216. doi:10.1056/NEJMoa2206038
7. Garvey WT, Frias JP, Jastreboff AM, et al. Tirzepatide once weekly for the treatment of obesity in people with type 2 diabetes (SURMOUNT-2): a double-blind, randomised, multicentre, placebo-controlled, phase 3 trial. *The Lancet*. 2023;402(10402):613-626. doi:10.1016/S0140-6736(23)01200-X
8. Wilding JPH, Batterham RL, Calanna S, et al. Once-Weekly Semaglutide in Adults with Overweight or Obesity. *New England Journal of Medicine*. 2021;384(11):989-1002. doi:10.1056/NEJMoa2032183
9. Davies M, Færch L, Jeppesen OK, et al. Semaglutide 2.4 mg once a week in adults with overweight or obesity, and type 2 diabetes (STEP 2): a randomised, double-blind, double-dummy, placebo-controlled, phase 3 trial. *The Lancet*. 2021;397(10278):971-984. doi:10.1016/S0140-6736(21)00213-0
10. Wilding JPH, Batterham RL, Davies M, et al. Weight regain and cardiometabolic effects after withdrawal of semaglutide: The STEP 1 trial extension. *Diabetes, Obesity and Metabolism*. 2022/08/01 2022;24(8):1553-1564. doi:https://doi.org/10.1111/dom.14725
11. Garvey WT, Batterham RL, Bhatta M, et al. Two-year effects of semaglutide in adults with overweight or obesity: the STEP 5 trial. *Nature Medicine*. 2022/10/01 2022;28(10):2083-2091. doi:10.1038/s41591-022-02026-4
12. Aronne LJ, Sattar N, Horn DB, et al. Continued Treatment With Tirzepatide for Maintenance of Weight Reduction in Adults With Obesity: The SURMOUNT-4 Randomized Clinical Trial. *JAMA*. 2024;331(1):38-48. doi:10.1001/jama.2023.24945
13. Weiss T, Carr RD, Pal S, et al. Real-World Adherence and Discontinuation of Glucagon-Like Peptide-1 Receptor Agonists Therapy in Type 2 Diabetes Mellitus Patients in the United States. *Patient Prefer Adherence*. 2020;14:2337-2345. doi:10.2147/ppa.S277676
14. Palanca A, Ampudia-Blasco FJ, Calderón JM, et al. Real-World Evaluation of GLP-1 Receptor Agonist Therapy Persistence, Adherence and Therapeutic Inertia Among Obese Adults with Type 2 Diabetes. *Diabetes Ther*. Apr 2023;14(4):723-736. doi:10.1007/s13300-023-01382-9

© 2025 Hwang JH et al. *JAMA Health Forum*.

15. Nguyen H, Dufour R, Caldwell-Tarr A. Glucagon-like peptide-1 receptor agonist (GLP-1RA) therapy adherence for patients with type 2 diabetes in a Medicare population. *Advances in therapy*. 2017;34:658-673.
16. Gleason PP, Urick BY, Marshall LZ, Friedlander N, Qiu Y, Leslie RS. Real-world persistence and adherence to glucagon-like peptide-1 receptor agonists among obese commercially insured adults without diabetes. *Journal of Managed Care & Specialty Pharmacy*. 2024/08/01 2024;30(8):860-867. doi:10.18553/jmcp.2024.23332
17. Do D, Lee T, Peasah SK, Good CB, Inneh A, Patel U. GLP-1 Receptor Agonist Discontinuation Among Patients With Obesity and/or Type 2 Diabetes. *JAMA Network Open*. 2024;7(5):e2413172-e2413172. doi:10.1001/jamanetworkopen.2024.13172
18. Kim DD, Wang L, Lauren BN, et al. Development and Validation of the US Diabetes, Obesity, Cardiovascular Disease Microsimulation (DOC-M) Model: Health Disparity and Economic Impact Model. *Med Decis Making*. Oct 16 2023:272989x231196916. doi:10.1177/0272989x231196916
19. Using Appropriate Price Indices for Expenditure Comparisons. Medical Expenditure Panel Survey. Accessed February 15, 2024, https://meps.ahrq.gov/about_meps/Price_Index.shtml
20. Ippolito BN, Levy JF. Estimating the Cost of New Treatments for Diabetes and Obesity. *AEI Economic Perspectives*. American Enterprise Institute; 2023. https://www.aei.org/wp-content/uploads/2023/09/Estimating-the-Cost-of-New-Treatments-for-Diabetes-and-Obesity.pdf
21. Hernandez I, Cousin EM, Wouters OJ, Gabriel N, Cameron T, Sullivan SD. Price benchmarks of drugs selected for Medicare price negotiation and their therapeutic alternatives. *J Manag Care Spec Pharm*. Jun 21 2024:1-11. doi:10.18553/jmcp.2024.24153
22. Dakin HA, Leal J, Briggs A, Clarke P, Holman RR, Gray A. Accurately Reflecting Uncertainty When Using Patient-Level Simulation Models to Extrapolate Clinical Trial Data. *Med Decis Making*. May 2020;40(4):460-473. doi:10.1177/0272989x20916442
23. Rubin DB. *Multiple Imputation for Nonresponse in Surveys*. Wiley-Interscience; 2004.
24. Benjamin EJ, Virani SS, Callaway CW, et al. Heart Disease and Stroke Statistics-2018 Update: A Report From the American Heart Association. *Circulation*. Mar 20 2018;137(12):e67-e492. doi:10.1161/CIR.0000000000000558
25. Centers for Disease Control and Prevention. Underlying Cause of Death 1999-2016. Accessed September 21, 2018. https://wonder.cdc.gov/wonder/help/ucd.html#
26. Lubetkin EI, Jia H, Franks P, Gold MR. Relationship among sociodemographic factors, clinical conditions, and health-related quality of life: examining the EQ-5D in the U.S. general population. *Qual Life Res*. Dec 2005;14(10):2187-96. doi:10.1007/s11136-005-8028-5
27. Davies EW, Matza LS, Worth G, et al. Health state utilities associated with major clinical events in the context of secondary hyperparathyroidism and chronic kidney disease requiring dialysis. *Health and Quality of Life Outcomes*. 2015/06/30 2015;13(1):90. doi:10.1186/s12955-015-0266-9
28. U.S. Centers for Medicare & Medicaid Services. Medicare Provider Utilization and Payment Data: Inpatient. 2019. https://www.cms.gov/Research-Statistics-Data-and-Systems/Statistics-Trends-and-Reports/Medicare-Provider-Charge-Data/Inpatient2017.html

© 2025 Hwang JH et al. *JAMA Health Forum*.
