# Supplementary material for: Fiscal Impact of Expanded Medicare Coverage for GLP-1 Receptor Agonists to Treat Obesity
Source: JAMA Health Forum. 2025 Apr 25;6(4):e250905. doi: 10.1001/jamahealthforum.2025.0905 (PMC12032556; doi:10.1001/jamahealthforum.2025.0905)
Supplement: Supplement 2. — Data Sharing Statement [file jamahealthforum-e250905-s002.pdf]

Data Sharing Statement

Hwang. Fiscal Impact of Expanded Medicare Coverage for GLP-1 Receptor Agonists to Treat Obesity. *JAMA Health Forum*. Published April 25, 2025.
doi:10.1001/jamahealthforum.2025.0905

Data
Data available: No

Additional Information
Explanation for why data not available: We used publicly available data.
